# Beneficial Effects of Cornelian Cherries on Lipid Profile and NO/ROS Balance in Obese Zucker Rats: Comparison with CoQ10

**DOI:** 10.3390/molecules25081922

**Published:** 2020-04-21

**Authors:** Ezgi Dayar, Martina Cebova, Jan Lietava, Elena Panghyova, Olga Pechanova

**Affiliations:** 1 Institute of Normal and Pathological Physiology, Centre of Experimental Medicine, Slovak Academy of Sciences, 841 04 Bratislava, Slovakia; ezgi.dayar@savba.sk (E.D.); martina.cebova@savba.sk (M.C.); jan.lietava@yahoo.com (J.L.); 2 1st Department of Internal Medicine, Medical Faculty of Comenius University, 811 07 Bratislava, Slovakia; 3 Research Institute of Nutrition, 821 08 Bratislava, Slovakia; panghyova@vup.sk

**Keywords:** cornelian cherry, CoQ10, lipid profile, nitric oxide, reactive oxygen species, aorta, heart, obese Zucker rat

## Abstract

Cornelian cherries (CCs) belong to promising anti-obesity substances. We aimed to study effects of coenzyme Q10 (CoQ10) and two varieties of CCs on lipid profile, ROS, and nitric oxide (NO) production in obese rats. Male Zucker rats were divided into the control group and groups treated with CoQ10 (30mg/kg/day), or CC varieties: Koralovij Marka (KM) and Wild Type (WT) (5 g/kg/day, *n* = 6 in each group) for 6 weeks. Blood pressure (BP), bodyweight, relative heart weight, and plasma lipid profile were determined. NOS activity and expressions of eNOS, SOD, and NADPH oxidase were determined in the left ventricle (LV) and aorta. Among CC groups, KM decreased bodyweight and WT relative heart weight. Neither CoQ10 nor CCs affected BP. CoQ10 did not affect lipid profile and NOS activity either in the LV or aorta. On the other hand, WT decreased cholesterol and LDL levels. KM and WT increased NOS activity in the aorta, while not affecting the activity in the LV. KM increased eNOS expression and did not affect ROS production, while WT increased SOD and decreased NADPH oxidase without affecting eNOS expressions in both tissues. In conclusion, CCs showed better beneficial effects than CoQ10 in all parameters studied.

## 1. Introduction

Metabolic syndrome is a clustering of known cardiovascular risk factors including obesity, hyperglycemia, insulin resistance, dyslipidemia, and hypertension [1,2]. There is a strong relationship between insulin resistance, visceral adiposity, and endothelial dysfunction. Each factor can contribute to the progression and development of each other [1]. Moreover, the risk factors of the metabolic syndrome can depend on genetic and environmental factors. Family history of type 2 diabetes, hypertension, insulin resistance, and ethnic background have greatly increased the risk of progression of metabolic syndrome [2]. The pathophysiology of metabolic syndrome is very complex and still unclear. Metabolic syndrome is associated with impairment of nitric oxide (NO) signalling and increasing the reactive oxygen species (ROS) generation which is related to the endothelial dysfunction [1,3]. Three approaches have been suggested to correct the balance between increased oxidative stress and decreased NO synthesis. First is reduction of ROS bioavailability via administration of different antioxidant compounds including polyphenol reach substances. Second is elevation of NO level by administration of NO donors such as nitroglycerin, nitrosothiols, or mono/dinitrates. And third, probably the most effective approach, is simultaneous ROS reduction and NO stimulation, e.g., by the treatment with angiotensin-converting enzyme inhibitors, AT1-receptor blockers, β-receptor blockers, or statins with NO-increasing properties [3,4,5,6,7]. Actually, statins belong to the first choice in the treatment of cardiovascular and obesity-associated diseases [5,7]. Under certain conditions, statin therapy may be however associated with residual risk and several side effects, e.g., locomotion disturbances, nonallergic rhinitis, hyperglycemia, or rhabdomyolysis [7]. Therefore, attention is also paid to the research of alternative treatments without harmful or side effects. 

Recently, many studies have pointed to the beneficial effects of polyphenolic substances contained in Cornelian cherry (*Cornus mas* L., CC). Cornelian cherry is a member of the *Cornaceae* family and known as a medicinal plant that grows in eastern and southern Europe, southwest Asia and Middle East [8,9,10]. All cultivars of the cornelian cherry have a high biological value, mainly associated with their antioxidant and anti-inflammatory activities which are attributed to a rich polyphenolic composition [11]. CC includes mainly anthocyanins, flavonoids, iridoids, phenolic acids, and tannins [8,12]. Actually, concentration of anthocyanins determines the color of fruits [8,11]. Except for polyphenols, CCs is famous for being a rich source of ascorbic acids, and essential minerals. CCs have a higher level of ascorbic acid than oranges and strawberries [8,13,14]. It also includes potassium and magnesium and in lower amount zinc, iron, copper, manganese, and sodium [8,12,13]. 

Recently it has been shown that CCs have anti-diabetic, anti-obesity, hypolipidemic and anti-atherosclerotic properties that were attributed to their anti-inflammatory and antioxidant effects [13,14]. In Wistar rats, hydroalcoholic fruits of CC were able to decrease blood glucose in a dose dependent manner [15]. In alloxan-induced diabetic rats, hydroalcoholic fruits of CCs also decreased triglycerides, very low-density lipoprotein (VLDL) and low-density lipoprotein (LDL) levels [16]. In the similar diabetes model, CC fruits effectively prevented the development of diabetes mellitus, increase of triglycerides and LDL, as well as elevation of aspartate, alanine aminotransferase, and alkaline phosphatase activities. Effects of CC fruits were comparable to that of glibenclamide [17]. On the other hand, in streptozotocin-induced diabetic rats, cornelian cherry dried powder was not able to normalise glucose level, however, it decreased cholesterol, LDL and increased high-density lipoprotein (HDL) levels and liver antioxidant capacity comparing the diabetic group. In the same model, cornelian cherry dried powder had a similar inhibitory effect on liver HMG-CoA reductase activity as lovastatin [18]. In New Zealand hypercholesterolemic rabbits, long-term treatment of CC powder decreased fibrinogen level even more significantly than lovastatin [19]. In high-fat diet mice, anthocyanins and ursolic acid extract from CCs improved glucose tolerance and decreased bodyweight gain by decreasing lipid accumulation [20]. Moreover, administration of CC powder in hypercholestrolemic rats were able to decrease triglycerides and had protective effects on atherosclerosis through enhanced PPARα protein expression and regulation of ROS production and inflammatory process [21]. 

The aim of our study was to investigate the effects of two varieties of CC, namely Koralovij Marka (KM) and Wild Type (WT) on lipid profile, blood pressure, reactive oxygen species (ROS) and nitric oxide (NO) production in obese Zucker rats. Moreover, the effects were compared with effective antioxidant—coenzyme Q10 (CoQ10). 

## 2. Results

### 2.1. Cornelian Cherry: Preparation and Characterisation

Wild Type of Cornelian cherries had about 3× higher content of total polyphenols, 2× higher antioxidant capacity and comparable concentration of total anthocyanidins comparing to Koralovij Marka (Table 1).

### 2.2. Bodyweight, Relative Heart Weight and Blood Pressure 

After six weeks of treatment, only KM group was able to decrease bodyweight in comparison to the control group. The relative heart weight (HW/TL) was significantly decreased in WT group only (Table 2). Treatment with CoQ10, KM, or WT did not affect blood pressure in obese Zucker rats (Table 2).

### 2.3. Plasma Lipid Profile

Plasma concentrations of total cholesterol and LDL were lower in WT group only compared to the control obese Zucker rats. Neither triglycerides nor HDL were changed within all groups (Table 3).

### 2.4. Total NOS Activity

**Figure 1:**
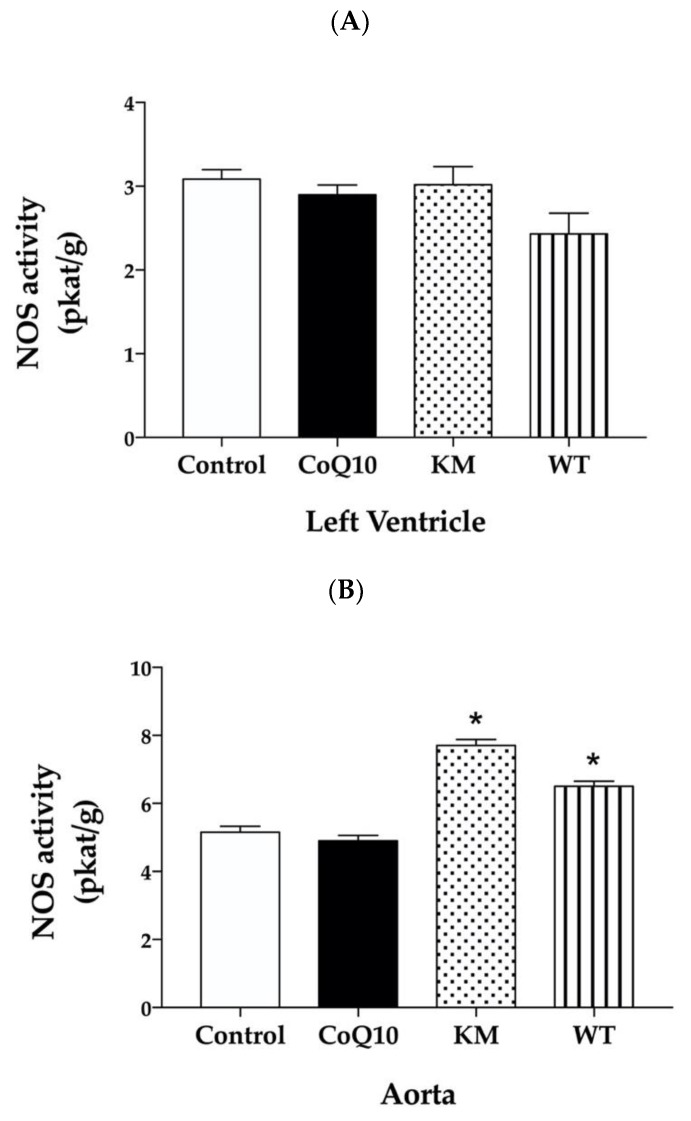
Effect of CoQ10, KM, and WT on total nitric oxide synthase (NOS) activity in the left ventricle (LV) (**A**) and aorta (**B**). CoQ10—coenzyme Q10, KM—Koralovij Marka, WT—Wild Type. Data are means ± SEM from 6 animals in each group. * *p* < 0.01 compared to the control group.

Total NOS activity in the left ventricle (LV) was not changed significantly within all groups (Figure 1A). On the other hand, CC varieties; KM and WT significantly increased NOS activity in the aorta (Figure 1B). CoQ10 did not affect NOS activity in both LV and aorta (Figure 1A,B). 

### 2.5. Protein Expressions of eNOS, NADPH Oxidase, and SOD 

**Figure 2:**
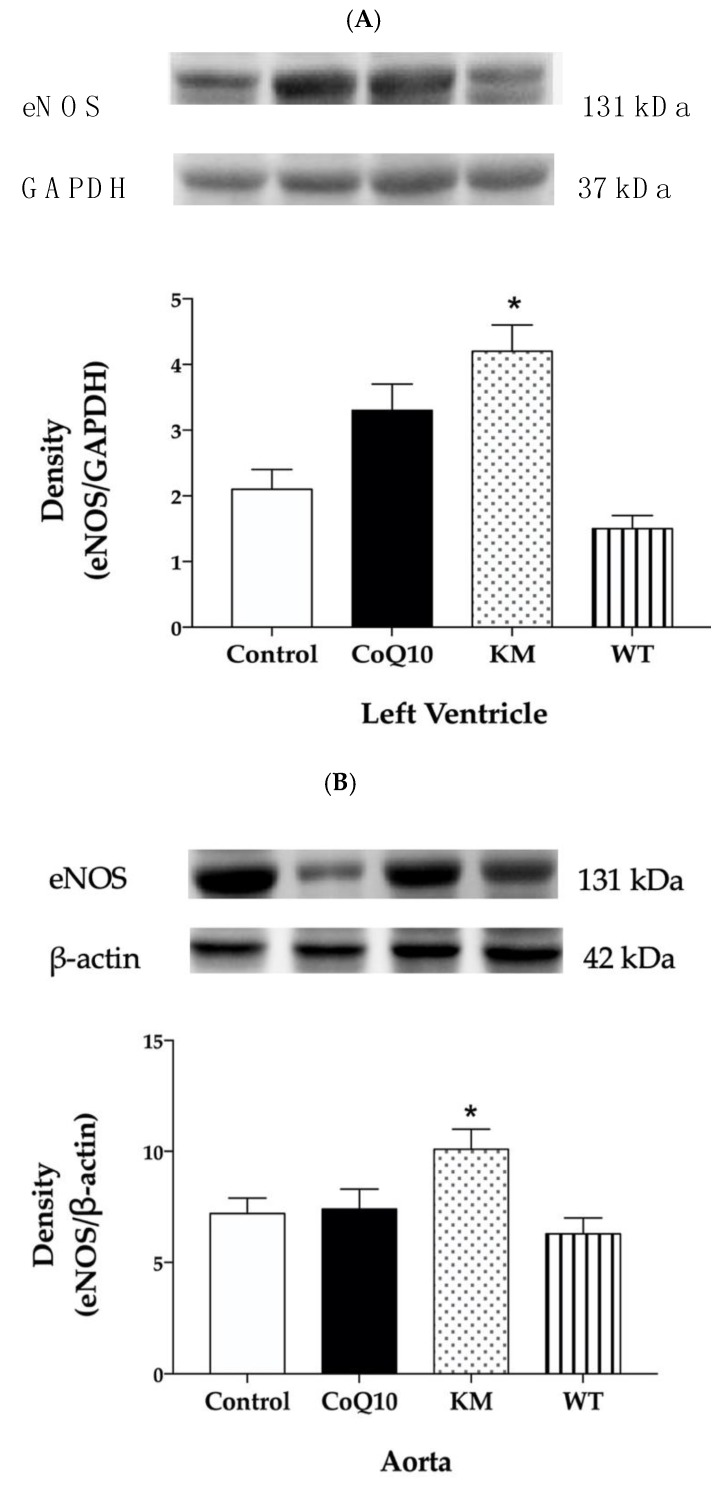
Effect of CoQ10, KM, and WT on eNOS protein expression in the left ventricle (LV) (**A**) and aorta (**B**). CoQ10—coenzyme Q10, KM—Koralovij Marka, WT—Wild Type. Data are means ± SEM from 6 animals in each group. * *p* < 0.05 compared to the control group.

**Figure 3:**
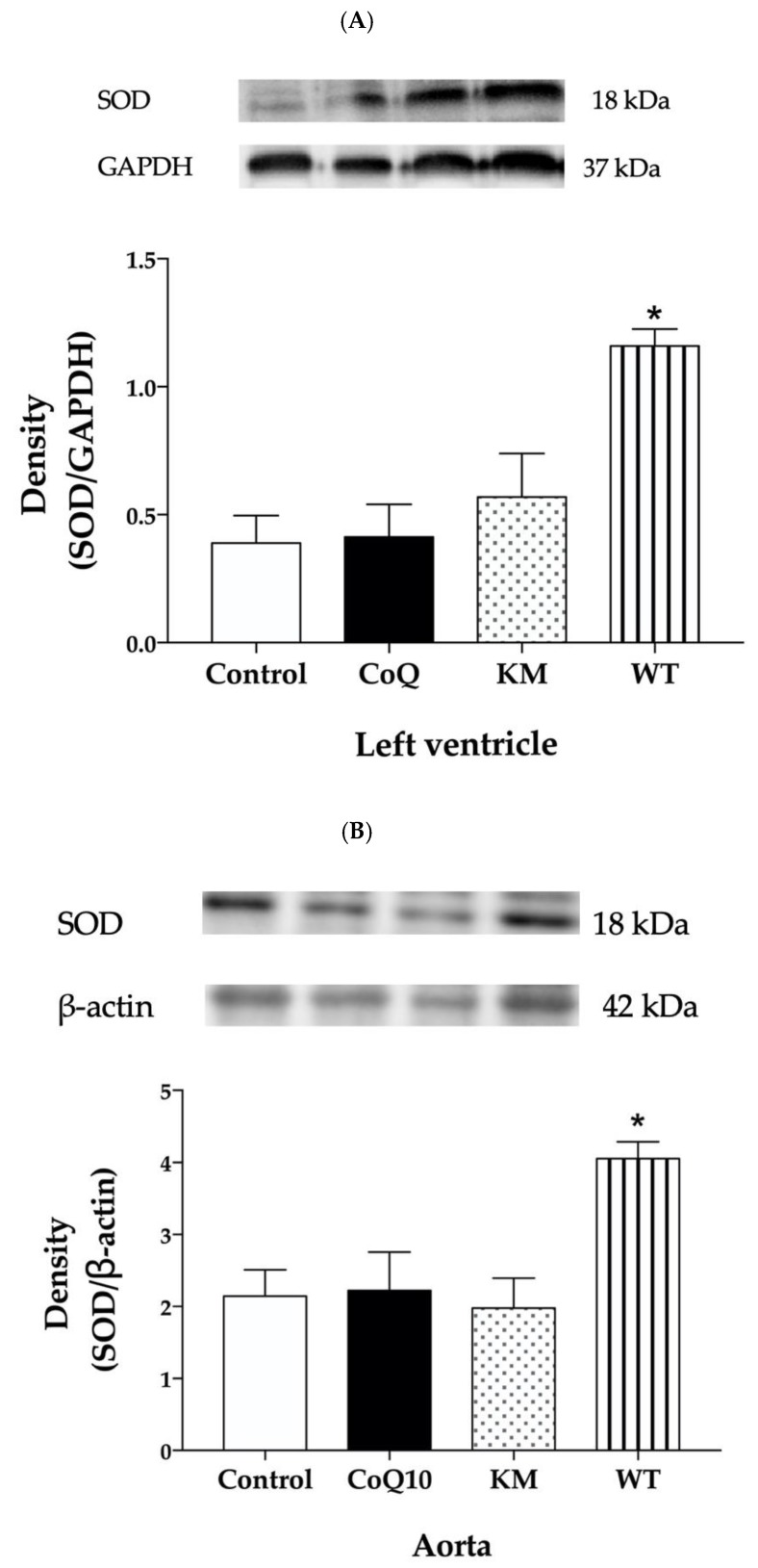
Effect of CoQ10, KM, and WT on SOD protein expression in the left ventricle (LV) (**A**) and aorta (**B**). CoQ10—coenzyme Q10, KM—Koralovij Marka, WT—Wild Type. Data are means ± SEM from 6 animals in each group. * *p* < 0.001 compared to the control group.

**Figure 4:**
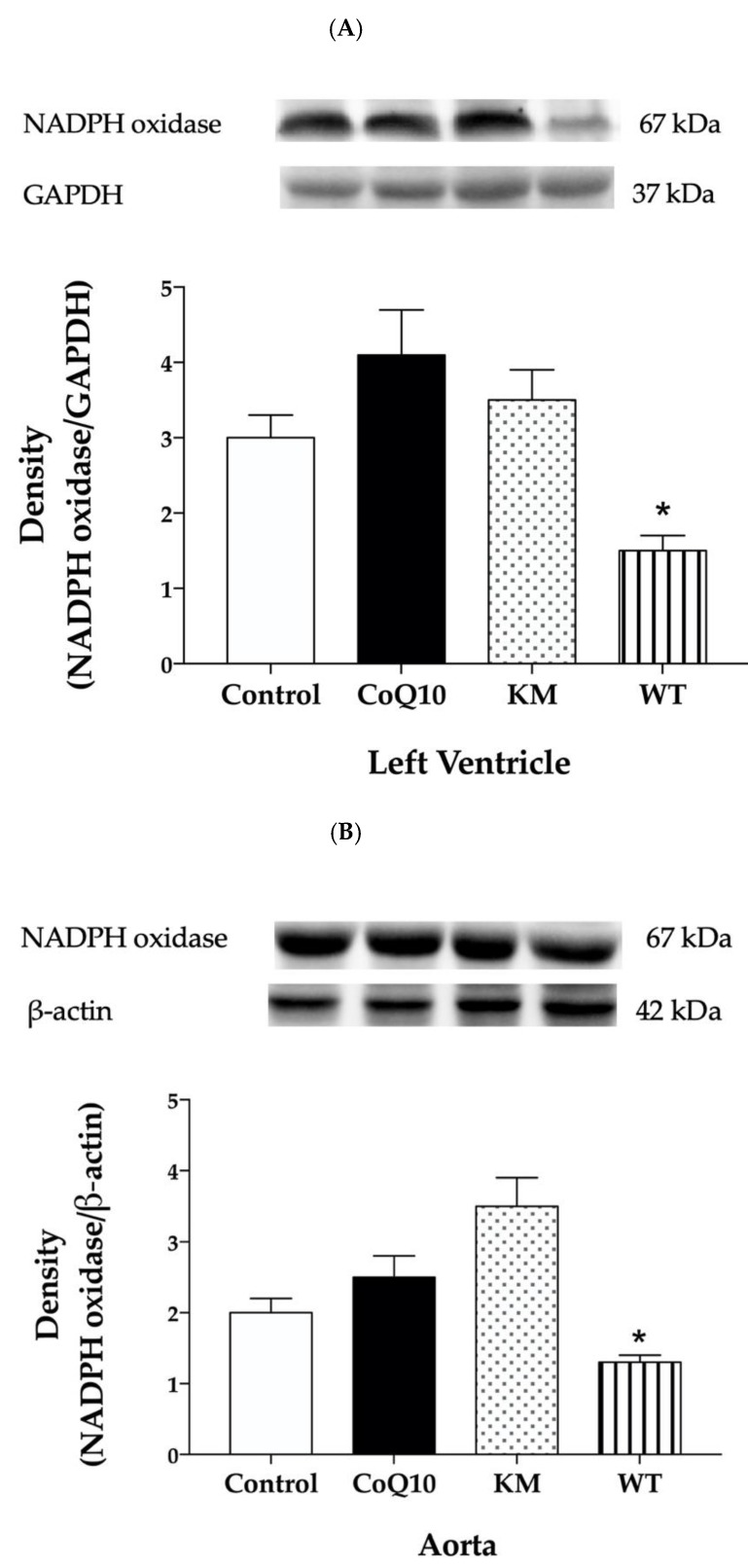
Effect of CoQ10, KM, and WT on NADPH oxidase protein expression in the left ventricle (LV) (**A**) and aorta (**B**). CoQ10—coenzyme Q10, KM—Koralovij Marka, WT—Wild Type. Data are means ± SEM from 6 animals in each group. * *p* < 0.05 compared to the control group.

Western blot analysis was used to determine protein expressions within all groups. KM treatment increased eNOS protein expression (Figure 2A,B) and did not affect SOD (Figure 3A,B) or NADPH oxidase (Figure 4A,B) expressions, while WT treatment increased SOD (Figure 3A,B) and decreased NADPH oxidase (Figure 4A,B) without affecting eNOS (Figure 2A,B) expressions in both left ventricle and aorta. CoQ10 did not affect expressions of any protein studied (Figure 2A,B, Figure 3A,B, and Figure 4A,B). 

### 2.6. Conjugated Diene Concentration

**Figure 5:**
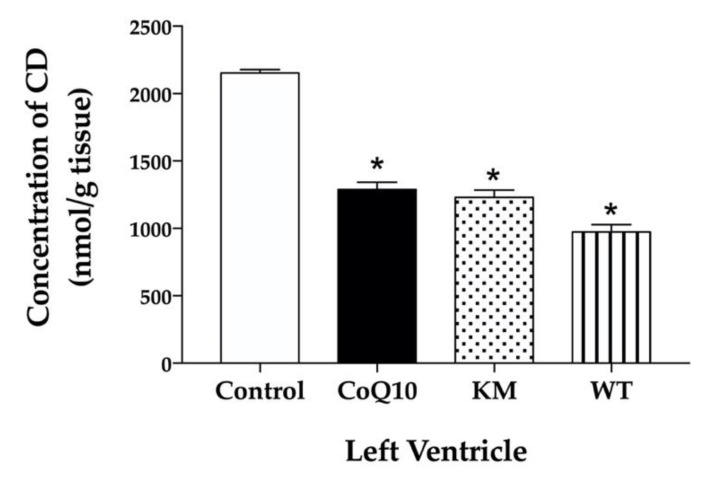
Effect of CoQ10, KM, and WT on concentration of conjugated dienes (CD) in the left ventricle. CoQ10; coenzyme Q10, KM; Koralovij Marka, WT; Wild Type. Data are means ± SEM from 6 animals in each group. * *p* < 0.001 compared to the control group.

All substances studied, namely CoQ10, KM and WT significantly and comparable decreased CD concentrations in the left ventricle (Figure 5). 

## 3. Discussion

The effects of two varieties of CC, namely Koralovij Marka and Wild Type, on lipid profile, blood pressure, reactive oxygen species, and nitric oxide production in obese Zucker rats were studied and compared with the effective antioxidant—coenzyme Q10. According to several studies, deficiency of CoQ10 is related to different diseases such as diabetes mellitus, atherosclerosis, hypertension, dyslipidemia, muscular dystrophy, and others. CoQ10 administration can protect against oxidative stress under conditions of cardiovascular disease, metabolic syndrome and type 2 diabetes [22]. Moreover, CoQ10 may decrease oxidised-LDL induced generation of ROS and improved antioxidant capacity. It also reduced oxidised-LDL-mediated downregulation of eNOS and upregulation of iNOS [23]. In our study, however, CoQ10 did not affect lipid profile and NOS activity either in the left ventricle or aorta. On the other hand, both KM and WT increased NOS activity in the aorta. While increased eNOS expression seems to be responsible for this effect in KM group, WT treatment probably stabilised NOS dimer by decreasing ROS production leading to increased NOS activity. 

The characterization of Cornelian Cherrie varieties showed that Wilde Type from Slovakia had about 3× higher content of total polyphenols, 2× higher antioxidant capacity and comparable concentration of total anthocyanidins comparing to Koralovij Marka from Ukraine. Higher number of polyphenols may be responsible for marked antioxidant capacity of Wilde Type. However, the antioxidant capacity may be increased also by the content of vitamin C, which has not been determined in the present study. According to the available literature, vitamin C content ranges from about 35 to 420 mg/100 in different CC and may vary according to the changing conditions [8,18]. A similar range of total polyphenols, as has been measured in our CC varieties, was determined in 10 samples of cornelian cherry fruits collected in Caucasus regions [24]. In the same study, total anthocyanin level varied from 11.2 to 92.2 mg/100g [18] and completely coincides with the level determined in our study (41.9 mg/100g in KM and 50.3 mg/100g in WT group).

Among CC groups, Koralovij Marka decreased bodyweight and WilC Type relative heart weight. Neither CoQ10 nor CCs affected BP. Similarly, supplementation of CC once a day in aged hamsters decreased weight gain and caused considerable hypoglycemic effect [25]. The anthocyanin extract from CC caused a 24% decrease in weight gain in high-fat-fed C57BL/6 mice. These mice also showed decreased lipid accumulation and triacylglycerol concentration in the liver [20]. In alloxan-induced diabetic rats, hydroalcoholic fruits of CCs in the dose 100 mg/kg for 110 days decreased triglycerides, VLDL and LDL levels [16]. In our study, plasma concentrations of total cholesterol and LDL were lower only in the WT group compared to the obese controls. Neither TG nor HDL were changed within all groups. 

We assume that decreased oxidative stress achieved by increased SOD and decreased NADPH oxidase protein expressions may be responsible for reduced cholesterol and LDL in WT group in our experiments. Similarly, Sozanski [21] reported protective effects of CC on diet-induced hypertriglyceridemia and atherosclerosis through regulation of oxidative stress in rabbits [21]. In another study, Sozanski et al. [26] documented that iridoids and anthocyanins from cornelian cherry fruits reduced formation of atherosclerotic plaques in the aorta of cholesterol-fed rabbits. Again, decreased oxidative stress determined as an increase of GSH and attenuated lipid peroxidation measured as malondialdehyde concentrations were responsible for this beneficial effect [26]. Similarly, Alavian et al. [27] showed reduced lipid peroxidation after CC treatment in CCl4-induced hepatotoxicity in rats [27]. Using a similar rat model, Es Haghi et al. [28] showed that treatment of rats with different doses of CC fruit extract (300 and 700 mg/kg) improved the alterations developed by CCl4 in lipid peroxidation, antioxidant defences, biochemical and renal lesions. In particular, CC fruit treatment increased serum SOD, catalase and glutathione peroxidase [28]. Our results are in good agreement with these studies since we found decreased concentration of another marker of lipid peroxidation and conjugated dienes after WT treatment in the heart as well. Interestingly, lipid peroxidation in the heart was reduced also after CoQ10 and KM treatments. Probably, another ROS regulating system, except SOD or NADPH oxidase, may be responsible for this effect. 

We also hypothesised that decreased oxidative stress after WT treatment stabilised NOS dimer and may be responsible for increased NOS activity in the aorta. KM treatment did not affect SOD or NADPH oxidase protein expression. However, it increased NOS activity as well. In this case, we hypothesised that increased protein expression of eNOS, found in the aorta, may contribute significantly to this effect. Similarly, treatment of rabbits with high-cholesterol diet with loganic acid or anthocyanins extracted from CC led to increased mRNA expression of eNOS in thoracic aortas. [29]. It seems that CC-derived anthocyanins represent promising molecules and dietary bioactive components that can effectively improve different factors of metabolic syndrome [30]. Previously, we have also reported that red wine polyphenolic compounds containing anthocyanins increased eNOS protein expression accompanied by increased NOS activity and improved vasorelaxation in L-NAME-hypertensive rats [31] or protected cardiac function in different models of metabolic syndrome [3]. 

Taken together, both varieties of CC—Koralovij Marka and Wild Type—increased NOS activity in the aorta of obese Zucker rats. While increased eNOS expression seems to be responsible for this effect in KM group, WT treatment probably stabilised NOS dimer and increased activity by decreasing ROS production via increased SOD and decreased NADPH oxidase protein expressions. It seems that other polyphenols, different from that involved in Koralovij Marka, are mostly responsible for antioxidant effect of Wild Type variety of Cornelian Cherry.

## 4. Materials and Methods

### 4.1. Chemicals

Most of the chemicals and reagents were obtained from Sigma-Aldrich (Saint-Louis, MO, USA); if not, the company is indicated.

### 4.2. Cornelian Cherry Preparation and Characterisation

Koralovij Marka were provided by the National Botanical Garden in Kiev, Ukraine. Wild Type originating in the White Carpathians, Slovakia. Both CCs were stored and dried under the same conditions. The fresh fruit was mixed with the standard feed and the addition of water so that the mixture was mouldable into the desired cuboid form 3 × 3 × 3 cm. Subsequently, the blocks were dried for 6 h at 50 °C to 90% dry weight on a tray dryer.

For determination of anthocyanins, total polyphenols and antioxidant activity, the stoned fruit was homogenised, and 15 g of fruit was extracted in 30 mL of acidified 70% ethanol for 30 min, the extraction was repeated until the extractant had decolorised.

The AOAC differential pH method [32] in two buffered solutions (KCL buffer pH 1.0 and sodium acetate buffer pH 4.5) was used to determine anthocyanin levels. The samples were diluted so that the absorbance of the solution was 0.2 to 1.2. After standing for 15 min in the dark, the absorbance at 510 and 700 nm was measured.

The total polyphenols content of the fruit extracts was determined by the Folin–Ciocalteu colorimetric method at 765 nm [33]. The total polyphenol content was calculated by the calibration curve method as an equivalent of gallic acid with a linearity of 100 to 800 mg/L, corresponding to an absorbance of 0.1–0.9 (*R*^2^ = 0.9954). The total polyphenols content was converted to the polyphenols content in fruits in mg/kg.

The antioxidant activity of the extracts was determined by the free radical DPPH method with an absorbance λ 0.8 at 516 nm [34] and calculated as a percentage of inhibition of the free radical DPPH. The EC 50 was calculated from the measured activities as the amount required to inhibit 50% DPPH in the reaction.

### 4.3. Animals and Treatment

All procedures and experimental protocols were approved by an Ethical committee of the Institute of Normal and Pathological Physiology Slovak Academy of Sciences (Ro-3601/17-221/3) according to the European Convention for the Protection of Vertebrate Animals used for Experimental and other Scientific Purpose, Directive 2010/63/EU of the European Parliament.

Obese Zucker (fa^−^/fa^−^) rats were obtained from Charles River, USA. They were housed in groups of 2 animals, under a 12 h light- 12 h dark cycle, at a constant humidity (45–65%) and temperature (20–22 °C).

Twelve-week-old male Zucker (fa^−^/fa^−^) rats were divided into the control group and groups treated with coenzyme Q10 and different varieties of CC namely: Koralovij Marka and Wild Type. Each group consisted of 6 animals. Control and CoQ10 groups were fed with a standard diet ad libitum, CC groups were fed with special diet which contained dry fruit of CC (5 g/kg/day) and mixed with standard diet (30 g/day). CoQ10 (30 mg/kg/day) was administered via the drinking water. The treatment lasted for 6 weeks. Daily water consumption was estimated individually for every animal and adjusted, if necessary. Blood pressure was measured noninvasively, using tail-cuff plethysmography weekly. At the end of the treatment, the animals were sacrificed; heart weight (HW) and tibia length (TL) were determined. Relative heart weight was calculated as a HW/TL ratio. Samples of left ventricle and aorta were used to determine NOS activity and eNOS, SOD and NADPH oxidase protein expressions. Lipid profile was analysed in the plasma and conjugated diene levels in the left ventricle.

### 4.4. Plasma Lipid Profile

The levels of triglyceride, total cholesterol, HDL and LDL were measured in the plasma by commercially available kits.

### 4.5. Total NOS Activity 

Total NOS activity was determined in crude homogenates of the LV and aorta by measuring the formation of [^3^H]-L-citrulline from [^3^H]-L-arginine (ARC, Saint Louise, MO, USA). 50 µL of 20% homogenates were incubated in the presence of 0.5 M Tris-HCl, pH 7.4, 10 mM NADPH, 20 mM CaCl_2_, 100 µM [^3^H]-L-arginine), 1mg/mL calmodulin, 1:1 FAD/FMN, and 50 mM TH_4_ in a total volume of 100 µL. After 30 min of incubation at 37 °C, the reaction was stopped by the addition of 1 mL of 0.02 M HEPES buffer pH 5.5, containing 2 mM EDTA, 2 mM EGTA, and 1 mM L-citrulline. The samples were then applied to 1 mL Dowex 50WX-8 columns (Na^+^ form). [^3^H]-L-citrulline was measured with Quanta Smart TriCarb Liquid Scintillation Analyser (Packard Instrument Company, Meriden, CT, USA).

### 4.6. Protein Expression Analysis by Western Blot

Protein expressions of eNOS, SOD, and NADPH oxidase were determined in the LV and aorta by Western blot analysis. The samples were probed with polyclonal rabbit anti-eNOS, anti-SOD and anti-NADPH oxidase antibodies and anti-GAPDH and anti β-actin as a loading control (Abcam, Cambridge, UK). The intensity of bands was visualised using the enhanced chemiluminescence system (ECL, Amersham, UK), quantified by using ChemiDocTM Touch Imagine System (Image Lab^TM^ Touch software, version 5.2, BioRad, Hercules, CA, USA) and normalised to GAPDH bands for LV and β-actin bands for aorta.

### 4.7. Conjugated Dienes Determination

The concentration of conjugated dienes was measured in lipid extracts of the LV. Samples of the LV were homogenised in 15 mmol/dm^3^ EDTA containing 4% NaCl. Lipids were extracted using a 1:1 chloroform-methanol mixture. Chloroform was evaporated in the N_2_ atmosphere and after the addition of cyclohexane, conjugated diene concentrations were determined spectrophotometrically (λ = 233 nm, NanpDrop 2000c, UV-Vis spectrophotometer). The concentration of CD was expressed as nmol per g tissue.

### 4.8. Statistics

The results are expressed as mean ± SEM. One-way analysis of variance (ANOVA) and Bonferroni test were used for statistical analysis. Values were considered significant with probability value *p* < 0.05 (both for ANOVA and Bonferroni test). The *p* values were multiplicity adjusted.

## 5. Conclusions

Our study has contributed significantly to the finding that cornelian cherries can be a significant functional food component with beneficial effects on cardiovascular and metabolic disorders. Moreover, for the first time, it has been shown that mechanisms of these beneficial effects may differ depending on the variety of cornelian cherry. We have demonstrated that the variety with higher content of total polyphenols had better antioxidant properties and that both varieties of cornelian cherry were able to increase NOS activity although by different mechanisms. It means that cornelian cherries have the ability to increase the production of endothelial NO and thus contribute to the improvement of cardiovascular function in metabolic syndrome. 

## Sample availability

Samples of the compounds are not available from the authors.

## Figures and Tables

**Table 1:** Determination of anthocyanins, total polyphenols and EC50 DPPH in stoned fruit.

Type	Total Polyphenols [mg/100 g]	Total Anthocyanidins [mg/100 g]	EC50 [g/L]
Koralovij Marka	151.2 ± 18.1	41.9 ± 5.0	0.54
Wild type	408.4 ± 49.0	50.3 ± 6.0	0.98

**Table 2:** Bodyweight (BW), heart weight (HW), heart weight (HW)/tibia length (TL) ratio and blood pressure (BP) in the control, coenzyme Q10 (CoQ10), Koralovij Marka (KM) and Wild Type (WT) groups.

	BW (g)	HW (g)	HW/TL (×10^−2^)	BP (mm-Hg)
Control	698.5 ± 20.4	1.33 ± 0.05	3.3 ± 0.1	147 ± 2.5
CoQ10	639.8 ± 42.3	1.29 ± 0.05	3.1 ± 0.1	142 ± 2.3
KM	611.5 ± 15.0 **	1.29 ± 0.02	3.2 ± 0.07	143 ± 5.4
WT	664 ± 10.4	1.15 ± 0.02 *	2.8 ± 0.03 **	137 ± 3.9

Data are means ± SEM from 6 animals in each group. ** *p* < 0.01 and * *p* < 0.05 compared to the control group.

**Table 3:** Lipid profile of control, coenzyme Q10 (CoQ10), Koralovij Marka (KM), and Wild Type (WT) groups.

	TG (mmol/L)	CHOL (mmol/L)	HDL (mmol/L)	LDL (mmol/L)
Control	2.87 ± 0.21	7.65 ± 0.18	147.3 ± 10.1	70.9 ± 2.7
CoQ10	2.91 ± 0.48	6.23 ± 0.52	143.2 ± 6.3	49.6 ± 4.1
KM	2.88 ± 0.42	6.17 ± 0.40	125.7 ± 3.9	46.9 ± 3.2
WT	2.77 ± 0.17	4.10 ± 0.33 *	128.3 ± 4.4	27.0 ± 1.3 *

TG; triglyceride, CHOL; total cholesterol, HDL; high-density lipoprotein, LDL; low-density lipoprotein. Data are means ± SEM from 6 animals in each group. * *p* < 0.001 compared to the control group.

## References

[1] Huang P.L. (2009). eNOS, metabolic syndrome and cardiovascular disease. Trends Endocrinol. Metab..

[2] Wong S.K., Chin K.Y., Suh F.H., Fairus A., Ima-Nirwana S. (2016). Animal models of metabolic syndrome: A review. Nutr. Metab. (Lond.).

[3] Pechánová O., Varga Z.V., Cebová M., Giricz Z., Pacher P., Ferdinandy P. (2015). Cardiac NO signalling in the metabolic syndrome. Br. J. Pharmacol..

[4] Münzel T., Gori T., Bruno R.M., Taddei S. (2010). Is oxidative stress a therapeutic target in cardiovascular disease?. Eur. Heart J..

[5] Cebova M., Rehakova R., Kosutova M., Pechanova O. (2018). Simvastatin does not affect nitric oxide generation increased by sesame oil in obese Zucker rats. Oxid. Med. Cell. Longev..

[6] Bagetta D., Maruca A., Lupia A., Mesiti F., Catalano R., Romeo I., Moraca F., Ambrosio F.A., Costa G., Artese A. (2020). Mediterranean products as promising source of multi-target agents in the treatment of metabolic syndrome. Eur. J. Med. Chem..

[7] Ramkumar S., Raghunath A., Raghunath S. (2016). Statin therapy: Review of safety and potential side effects. Acta Cardiol. Sin..

[8] Dinda B., Kyriakopoulos A.M., Dinda S., Zoumpouris V., Thomaidis N.S., Velegraki A., Markopoulos C., Dinda M. (2016). *Cornus mas* L. (cornelian cherry), an important European and Asian traditional food and medicine: Ethnomedicine, phytochemistry and pharmacology for Its commercial utilization in drug industry. J. Ethnopharmacol..

[9] Gholamrezayi A., Yaghubi E., Ghafouri A. (2019). A review of probable effects of cornelian cherry fruit. J. Biochem. Tech..

[10] Szczepaniak O.M., Kobus-Cisowska J., Kusek W., Przeor M. (2019). Functional properties of Cornelian cherry (*Cornus mas* L.): A comprehensive review. Eur. Food Res. Technol..

[11] Kazimierski M., Regula J., Molska M. (2019). Cornelian cherry (*Cornus mas* L.)—characteristics, nutritional and pro-health properties. Acta Sci. Pol. Technol. Aliment..

[12] Gąstoł M., Krośniak M., Derwisz M., Dobrowolska-Iwanek J. (2013). Cornelian cherry (*Cornus mas* L.) juice as a potential source of biological compounds. J. Med. Food.

[13] Lietava J., Beerova N., Klymenko S.V., Panghyova E., Varga I., Pechanova O. (2019). Effects of Cornelian cherry on atherosclerosis and its risk factors. Oxid. Med. Cell. Longev..

[14] Hassanpour H. (2011). Antioxidant capacity and phytochemical properties of cornelian cherry (*Cornus mas* L.) genotypes in Iran. Sci. Hortic..

[15] Narimani-Rad M., Zendehdel M., Abbasi M.M., Abdollahi B., Lotfi A. (2013). Cornelian cherry (*Cornus mas* L.) extract affects glycemic status in Wistar rats. Bull. Environ..

[16] Mirbadalzadeh R., Shirdel Z. (2012). Antihyperglycemic and antihyperlipidemic effects of *Cornus mas* extract in diabetic rats compared with glibenclamide. Horm. Signal..

[17] Asgary S., Rafieian-Kopaei M., Shamsi F., Najafi S., Sahebkar A. (2014). Biochemical and histopathological study of the anti-hyperglycemic and anti-hyperlipidemic effects of cornelian cherry (*Cornus mas* L.) in alloxan-induced diabetic rats. J. Complement. Integr. Med..

[18] Hosseinpour-Jaghdani F., Shomali T., Gholipour-Shahraki S., Rahimi-Madiseh M., Rafieian-Kopaei M. (2017). *Cornus mas*: A review on traditional uses and pharmacological properties. J. Complement. Integr. Med..

[19] Asgary S., Rafieian-Kopaei M., Adelnia A., Kazemi S., Shamsi F. (2010). Comparing the effects of lovastatin and *Cornus mas* fruit on fibrinogen level in hypercholesterolemic rabbits. ARYA Atheroscler. J..

[20] Jayaprakasam B., Olson L.K., Schutzki R.E., Tai M.H., Nair M.G. (2006). Amelioration of obesity and glucose intolerance in high-fat-fed C57BL/6 mice by anthocyanins and ursolic acid in Cornelian cherry (*Cornus mas*). J. Agric. Food Chem..

[21] Sozanski T., Kucharska A.Z., Szumny A., Magdalan J., Bielska K., Merwid-Lad A., Wozniak A., Dzimira S., Piorecki N., Trocha M. (2014). The protective effect of the *Cornus mas* fruits (cornelian cherry) on hypertriglyceridemia and atherosclerosis through PPARα activation in hypercholesterolemic rabbits. Phytomedicine.

[22] Potgieter M., Pretorius E., Pepper M.S. (2013). Primary and secondary coenzyme Q10 deficiency: The role of therapeutic supplementation. Nutr. Rev..

[23] Tsai K.L., Huang Y.H., Kao C.L., Yang D.M., Lee H.C., Chou H.Y., Chen Y.C., Chiou G.Y., Chen L.H., Yang Y.P. (2012). A novel mechanism of coenzyme Q10 protects against human endothelial cells from oxidative stress-induced injury by modulating NO-related pathways. J. Nutr. Biochem..

[24] Perova I.B., Zhogova A.A., Poliakova A.V., Éller K.I., Ramenskaia G.V., Samylina I.A. (2014). [Biologically active substances of cornelian cherry fruits (*Cornus mas* L.)]. Vopr. Pitan..

[25] Rasoulian H., Shahryar H.A., Abbaspour R., Lotfi H. (2012). Effects of dietary inclusion of cornelian cherry (*Cornus mas* L.) fruit on body weight, insulin level and glycemic status of hamsters. Pak. J. Biol. Sci..

[26] Sozański T., Kucharska A.Z., Dzimira S., Magdalan J., Szumny D., Matuszewska A., Nowak B., Piórecki N., Szeląg A., Trocha M. (2018). Loganic acid and anthocyanins from cornelian cherry (*Cornus mas* L.) fruits modulate diet-induced atherosclerosis and redox status in rabbits. Adv. Clin. Exp. Med..

[27] Alavian S.M., Banihabib N., Es Haghi M., Panahi F. (2014). Protective Effect of *Cornus mas* Fruits Extract on Serum Biomarkers in CCl4-Induced Hepatotoxicity in Male Rats. Hepat. Mon..

[28] Es Haghi M., Dehghan G., Banihabib N., Zare S., Mikaili P., Panahi F. (2014). Protective effects of *Cornus mas* fruit extract on carbon tetrachloride induced nephrotoxicity in rats. Indian J. Nephrol..

[29] Sozański T., Kucharska A.Z., Wiśniewski J., Fleszar M.G., Rapak A., Gomułkiewicz A., Dzięgiel P., Magdalan J., Nowak B., Szumny D. (2019). The iridoid loganic acid and anthocyanins from the cornelian cherry (*Cornus mas* L.) fruit increase the plasma l-arginine/ADMA ratio and decrease levels of ADMA in rabbits fed a high-cholesterol diet. Phytomedicine.

[30] Gowd V., Jia Y., Chen W. (2017). Anthocyanins as promising molecules and dietary bioactive components against diabetes. A review of recent advances. Trends Food Sci. Technol..

[31] Pechánová O., Bernátová I., Babál P., Martínez M.C., Kyselá S., Stvrtina S., Andriantsitohaina R. (2004). Red wine polyphenols prevent cardiovascular alterations in L-NAME-induced hypertension. J. Hypertens..

[32] Mazza G., Cacace J.E., Kay C.D. (2004). Methods of analysis for anthocyanins in plants and biological fluids. AOAC Int..

[33] Singleton V.L., Orthofer R., Lamuela-Raventós R.M. (1999). Polyphenols and flavonoids: Analysis of total phenols and other oxidation substrates and antioxidants by means of Folin-Ciocalteu Reagent. Methods Enzym..

[34] Brand-Williams W., Cuvelier M.E., Berse C. (1995). Use of a Free Radical Method to Evaluate Antioxidant Activity. LWT-Food Sci. Technol..

